# Prehatch Calls and Coordinated Birth in Turtles

**DOI:** 10.1002/ece3.70410

**Published:** 2024-10-22

**Authors:** Gabriel Jorgewich‐Cohen, Madeleine Wheatley, Lucas Pacciullio Gaspar, Peter Praschag, Nicole Scholte Lubberink, Keesha Ming, Nicholas A. Rodriguez, Camila R. Ferrara

**Affiliations:** ^1^ Department of Palaeontology University of Zurich Zurich Switzerland; ^2^ Department of Evolutionary Anthropology University of Zurich Zurich Switzerland; ^3^ Turtle Island – Turtle Conservation and Research Centre Graz Styria Austria; ^4^ Departamento de Biodiversidade, Laboratório de Ecologia Espacial e conservação (LEEC) Universidade Estadual Paulista Julio de Mesquita Filho, Campus Rio Claro Rio Claro São Paulo Brazil; ^5^ School of Biological Sciences The University of Western Australia Perth Western Australia Australia; ^6^ Wildlife Conservation Society – WCS Brasil Manaus Amazonas Brazil

**Keywords:** acoustic repertoire, nest emergence, synchrony, vocalisation

## Abstract

Hatching synchronisation is widespread in oviparous taxa. It has been demonstrated that many species use sounds to coordinate synchronous hatching, being widespread among archosaurs (birds and crocodilians). Recent studies have shown that some turtle species produce vocalisations from within the egg, but the role of this behaviour in synchronising hatch is untested. The small amount of information about sound production by turtle embryos, limited to a handful of closely related species, precludes any inferences based on differences in their ecology, reproductive behaviour and phylogenetic context. With the goal to investigate if coordinated synchronous behaviour is mediated by within‐egg vocalisations in turtles, we recorded clutches from six different turtle species. The selected animals present different ecological and reproductive niches and belong to distinct phylogenetic lineages at the family level. We aimed to understand: (1) what is the phylogenetic distribution of within‐egg vocal behaviour among turtles; (2) if asynchronous turtle species vocalise from within the egg; (3) if clutch size influences synchronous behaviour and (4) if within‐egg turtle calls follow any phylogenetic signal. The new evidence provides light to the current knowledge about synchronous behaviour and within‐egg calls, challenging previous hypothesis that within‐egg sounds are accidentally produced as side‐effects of other behaviours.

## Graphical abstract

The study investigates the phylogenetic distribution of within‐egg vocal behaviour among turtles and its role in synchronous hatching. Clutches from six turtle species with diverse ecological and reproductive niches were recorded, aiming to understand if asynchronous species vocalise from within the egg, the influence of clutch size on synchronous behaviour and the presence of any phylogenetic signal in within‐egg turtle calls. The findings challenge previous hypotheses, suggesting that within‐egg sounds are not accidental side‐effects but may indeed mediate coordinated synchronous behaviour in turtles.

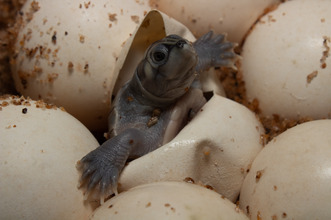

## 1 Introduction

Hatching synchronisation is widespread in oviparous taxa, being found in insects (Endo and Numata 2020), fish (Majoris et al. 2022), amphibians (Warkentin 2011), turtles (Spencer and Janzen 2011), archosaurians (Ferguson 1985; Vergne and Mathevon 2008; Mariette, Clayton, and Buchanan 2021) and squamates (Aubret et al. 2016); it may have evolved recurrently in oviparous lineages. It acts in different forms and intensities (Colbert, Spencer, and Janzen 2010), that can vary up to the population level (McGlashan et al. 2018). Although seemingly widespread, the evolutionary drivers for the selection of synchronous hatching are not well understood and seem to vary among animals in different ecological contexts (Riley et al. 2020).

Most of the knowledge about synchronous hatching behaviour comes from archosaurians (birds and crocodilians; e.g., Ferguson 1985; Vergne and Mathevon 2008; Mariette, Clayton, and Buchanan 2021), which are some of the most studied animals due to the high number of social behaviours they display. In fact, synchronisation has been hypothesised to be linked to highly social behaviours such as parental care and vocal communication: prehatch vocalisations are used by birds (Brua 2002; Mariette and Buchanan 2016; Noguera and Velando 2019) and crocodilians (Magnusson 1980; Vergne and Mathevon 2008) to mediate synchronous hatching. Furthermore, synchronised hatching facilitates parental care, as incubation and feeding/protection of hatchlings do not happen concurrently (Vergne and Mathevon 2008; Mariette and Buchanan 2016; but see Węgrzyn, Węgrzyn, and Leniowski 2023). Birds may also synchronise hatching in order to avoid less‐favourable conditions after hatching of the first eggs (Mariette and Buchanan 2016) and both birds and crocodilians communicate with their parents from within the egg (Brua, Nuechterlein, and Buitron 1996; Vergne et al. 2007)—which may even be involved in vocal learning in some birds (Katsis et al. 2018; Colombelli‐Négrel et al. 2021).

Differently from archosaurs, turtles mostly lack parental care, limited to temporary nest protection in a handful of species (Barrett and Humphrey 1986; Iverson 1990; Kuchling 1999). The South American river turtle (*Podocnemis expansa*) is currently the only turtle species thought to display post hatch parental care (Ferrara, Vogt, and Sousa‐Lima 2012). Many researchers advocate that synchrony in hatching behaviour is associated to the lack of parental care (e.g., Jarrett et al. 2018; Pearson and Warner 2018). Hatchlings can benefit from synchronous birth by sharing the burden of digging out of the nest (Rusli, Booth, and Joseph 2016) and decrease individual chances of being predated by swamping predators (Arnold and Wassersug 1978; Ims 1990; Santos et al. 2016). Predation pressure might also have had a role in the selection of synchronous hatching, as eggs that hatch late would get exposed once the first individuals leave the nest (McGlashan et al. 2018).

Furthermore, in the last decade, turtles have been recognised as vocal animals, with all studied species—around one‐third of the clade—being recorded producing sounds (Ferrara et al. 2013; Jorgewich‐Cohen, Townsend, et al. 2022). Likewise, sound production from within the eggs and nests has been reported in some species such as all sea turtles (Ferrara, Mortimer, et al. 2014; Ferrara, Vogt, et al. 2014; Ferrara et al. 2019; Monteiro et al. 2019; McKenna et al. 2019; Field 2020; Nishizawa et al. 2021; Jorgewich‐Cohen, Townsend, et al. 2022), three river turtles (*Podocnemis* spp.; Ferrara, Vogt, and Sousa‐Lima 2012; Del Río 2022), one map turtle (*Graptemys ouchatensis*; Geller and Casper 2019a), one softshell turtle (*Apalone spinifera*; Geller and Casper 2023) and the common snapping turtle (*Chelydra serpentina*, Geller and Casper 2019b; Lacroix, Davy, and Rollinson 2022).

Considering that turtles likely represent the sister clade to birds and crocodilians (Joyce et al. 2021), it is reasonable to deem similar ecological value to the within‐egg vocalisations produced by these animals. The discovery of within‐nest acoustically mediated interaction in turtles has opened the discussion about the role of such signals, and the possibility that sounds are used to synchronise hatch (Ferrara, Vogt, and Sousa‐Lima 2012; Ferrara, Mortimer, et al. 2014; Ferrara, Vogt, et al. 2014; Ferrara et al. 2019; Geller and Casper 2019a; Doody, Dinets, and Burghardt 2021—but see McKenna et al. 2019; Lacroix, Davy, and Rollinson 2022). Furthermore, the distinct absence of parental care and the diversity of ecological niches occupied by turtles make them a great model to study prehatch vocalisations and its potential links to synchronous hatching.

Synchronised hatching behaviour has only been studied in half dozen turtle species (Spencer, Thompson, and Banks 2001; Colbert, Spencer, and Janzen 2010; Spencer 2012; Doody et al. 2012; McGlashan, Spencer, and Old 2012; McGlashan et al. 2015, 2017; Riley et al. 2020; Field, McGlashan, and Salmon 2021; Bock et al. 2022; Lacroix, Davy, and Rollinson 2022), and the strategies used to achieve it have been shown to be diverse. Synchronous behaviours can be divided into four not necessarily mutually excluding categories: (1) *temporal synchrony* is induced by maternal effects that impose constrained incubation periods (Ims 1990; Aubret et al. 2016). Although it influences the time of egg incubation, it is not mediated by embryos coordination. Synchronicity can also be achieved through (2) *environmental synchrony*, where ecological cues induce hatching (Doody 2011). This can be observed in the pig‐nose turtle (*Carettochelys insculpta*), where embryos emerge after being subjected to hypoxia caused by nest flooding (Doody et al. 2012). This strategy also does not necessarily require any sort of embryo‐embryo communication.

The necessity of coordination among hatchlings and embryos has been reported in synchronised digging behaviour (Houghton and Hays 2001; Rusli and Booth 2016), which may represent a case of (3) *apparent synchrony* (or emergence synchrony), where hatching does not happen at the same time, but the first‐born waits in the nest for their siblings to hatch (McGlashan et al. 2018), and only nest emergence is synchronised. True hatch synchrony, or (4) *coordinated synchrony*, happens when hatchlings communicate their developmental status to their siblings, which alter the time periods of incubation through physiological mechanisms in order to hatch at a similar time—despite potential thermal differences in the nest (Ims 1990; McGlashan, Spencer, and Old 2012; Aubret et al. 2016). Hypothetically, there are three ways in which coordinated synchrony can happen: (a) ‘*catch up*’, where embryos subjected to lower temperatures—that is, less developed—increase their developmental rates so that they can hatch at a synchronised time with more developed clutch mates (e.g., *Emydura, Chelodina* and *Apalone*; Spencer, Thompson, and Banks 2001; McGlashan et al. 2012; Riley et al. 2020); (b) *delayed hatch*, in which embryos aestivate and eggs do not hatch although they are completely developed or they stop developing at certain stage to wait for their siblings or better weather condition (Doody 2011); and (c) *early hatch*, where not yet fully developed eggs simply hatch following their siblings (e.g., *Chelydra* and *Chrysemys*; Spencer and Janzen 2011; McGlashan et al. 2018; Riley et al. 2020; Lacroix, Davy, and Rollinson 2022).

The physiological costs associated with synchronised hatching indicate that this behaviour has adaptative value (McGlashan et al. 2018; Riley et al. 2020). Together with the fact that vocalisations are widely used by archosaurs in within‐nest communication (Brua, Nuechterlein, and Buitron 1996; Vergne et al. 2007), it is parsimonious to infer communicative meaning to similar vocal behaviours in turtles. Yet, the limited information about turtle within‐nest vocalisations makes it hard to understand patterns based on the phylogenetic distribution of this behaviour. Moreover, the species so far reported to vocalise prior birth have similar reproductive strategies (Jorgewich‐Cohen, Henrique, et al. 2022), with large clutches and synchronised hatch—which can be expected to shape vocal behaviour. Information on species that lay one or few eggs that do not synchronise hatch would bring light to the discussion about the adaptative value and use of within‐egg vocalisations by turtles.

With the goal of investigating if coordinated synchronous behaviour is mediated by within‐egg vocalisations in turtles, we recorded clutches from different turtle species. The selected animals present different ecological and reproductive niches and belong to distinct phylogenetic lineages at the family level. We aimed to inspect: (1) what is the phylogenetic distribution of within‐egg vocal behaviour among turtles; (2) if asynchronous turtle species vocalise from within the egg; (3) if clutch size influences prehatch calls and synchronous behaviour and (4) if within‐egg turtle calls follow any phylogenetic signal. The new evidence provides light on the current knowledge about synchronous behaviour and within‐egg calls.

## 2 Methods

Nests from six different turtle species were recorded from the final 6 days of incubation to hatching day. We conducted experiments in the field and in captivity.

### 2.1 Species

Species selection was subjected to the availability of nests, but aimed to include representatives of all major turtle clades (Table 1). We also selected species with different reproductive strategies regarding clutch size (Jorgewich‐Cohen, Henrique, et al. 2022) that are expected to present different patterns of synchronous hatching behaviour: from 1 to 4 eggs, from 5 to 29 eggs and 30 or more eggs. We included the South American river turtle (*Podocnemis expansa*) as a control species, since it is already known to vocalise from within the egg (Ferrara, Vogt, and Sousa‐Lima 2012).

**TABLE 1:** Species selected for the present study.

Species	Family	Clutch size	Source
*Podocnemis expansa*	Podocnemididae	Up to 130	In situ
*Chitra indica*	Trionychidae	Up to 200	Captive
*Pseudemydura umbrina*	Chelidae	3–5	Mixed
*Kinosternon subrubrum*	Kinosternidae	2–5	Captive
*Batagur baska*	Geoemydidae	15–30	Captive
*Deirochelys reticularia*	Emydidae	4–10	Captive

### 2.2 Recordings

A professional recorder Tascam (dr‐100 mk iii) with 192 kHz/24‐bit resolution was used in combination with an omnidirectional microphone (Rode—Lavalier Go) for egg recording. The microphone was positioned among the eggs (detailed setup information and photos can be found in Appendix S1).

Estimated hatching dates were calculated based on the known incubation period of each species. Clutches were recorded every day averaging between 7 and 8 h a day, starting 2 weeks prior expected hatch date in order to ensure that the last days of development—where sound production is known in other species (Brua 2002; Vergne and Mathevon 2008)—would not get lost due to early hatch. We analysed the recordings starting from 6 days prior hatching date until a day after hatch.

### 2.3 Recordings in Captivity

Most recordings were conducted in captivity at Turtle Island, Styria, Austria. *Pseudemydura umbrina* eggs were recorded at Perth Zoo, Australia. We had access to one clutch from each species, except for *P. umbrina*, of which we analysed five clutches. Eggs from the same clutch were incubated together and placed 1 cm from each other in all trials.

### 2.4 Recordings In Situ

Field recordings were conducted at the Trombetas River Biological Reserve, Pará, Brazil, where ten nests of *Podocnemis expansa* were recorded for an average of 40 min each. Nests were oviposited approximately at the same date, and hatched a few days after recordings were conducted. Additionally, approximately 8 h of recordings were conducted in one wild nest of *P. umbrina* at Ellen Brook Nature Reserve, Perth, Australia. This nest was oviposited on November 14, 2020, and the recording was conducted on April 28, 2021—approximately 1 week before nest emergence. The microphone was inserted in the nest, where eggs were positioned as laid. In comparison to recordings in captivity, wild nests were not exhaustively analysed due to time constraints.

### 2.5 Analyses of Acoustic Repertoires

We used Raven Pro 1.6 (Cornell Lab of Ornithology, Ithaca, NY, 2023) to analyse the recordings and search for sounds produced by embryos. The software R version 4.2.3 (R Core Team 2022) was used to cut and measure sound parameters of sounds based on their aural and spectral characteristics. Sounds were categorised following traits used in previous research describing turtle acoustic repertoires (Ferrara et al. 2013; Lacroix, Davy, and Rollinson 2022): dominant frequency, maximum and minimum frequency, sound duration, mean variations of the intensity contour and number of pulses.

We chose for a conservative description of the vocal repertoire in order to assure we are only including sounds produced by the species. Therefore, we excluded any sounds that had an ambiguous source (i.e., not obviously produced by the turtles). Sounds were sorted into different categories based on human perception, using acoustic and visual cues based on the aural and spectral characteristics of the vocalisations.

### 2.6 Phylogenetic Distribution of Prehatch Calls and Synchronous Birth in Turtles

We compiled information about turtle species that have had their nests recorded in search of acoustic behaviour and species that have been studied regarding synchronous hatch. This information was then plotted in a phylogenetic tree with character states that represent absence and presence of these behaviours: (1) within‐egg calls (0, absent; 1, present; 2, not recorded) and (2) synchronous behaviour (0, absent; 1, present; 2, apparently absent; 3, apparently present). Character states assigned to each turtle species can be found in Appendix S2. Additionally, we performed an ancestral‐state reconstruction analysis for the presence or absence of both synchronous behaviour and prehatch call—which was inferred for each ancestral node in the tree using maximum‐likelihood reconstruction.

We used an edited version of the phylogeny proposed by Pereira et al. (2017). The tree was pruned using the function drop. tip from the Ape package (Paradis and Schliep 2019) in R platform (R Core Team 2022). We created a tree containing only the taxa to which some information about vocal and/or synchronous behaviours were available and used it to analyse the distribution of these traits among turtles.

### 2.7 Correlations Among Prehatch Calls, Synchronous Birth and Ecological Traits

In order to understand if there are any correlations between prehatch calls and synchronous behaviour and if they correlate to clutch size in a phylogenetic context, we performed a phylogenetic principal component analysis (phyPCA). Additionally, we included information from previous studies about other ecological traits that may influence vocal and synchronous behaviours: eggshell structure (hard or soft‐shelled), mean incubation time, nest depth (Field, McGlashan, and Salmon 2021), presence or absence of diapause during incubation (Ewert 1991; Horne 2007), and type of sex determination (genetic or temperature determined; Bista and Valenzuela 2020). We used the function phyl.pca (package phytools; Revell 2012) in the R platform.

### 2.8 Test Phylogenetic Signal of Within‐Egg Turtle Calls

To test if there is any phylogenetic signal in within‐egg turtle sounds, we used all known sound types from all species recorded in this study and in previous studies that were available to us. These include *Podocnemis expansa* and *Batagur baska* (present work), *Chelydra serpentina*, *Graptemys ouachitensis* and *Apalone spinifera* (Geller and Casper 2019a, 2019b, 2023, respectively), all sea turtles (Ferrara, Mortimer, et al. 2014; Field 2020; Jorgewich‐Cohen, Townsend, et al. 2022)—except for *Eretmochelys imbricata* and *Lepidochelys olivacea* as we were unable to access samples.

We used one sound sample of each kind from each species. Sounds were resampled to the same sampling rate and bit depth using Audacity, and their characters were extracted using the spectro_analysis function of the package warbleR (Araya‐Salas and Smith‐Vidaurre 2017). We ran a PCA using the extracted parameters and plotted the information from the first two PCs in order to visualise the similarity among sounds. Those that were plotted closer were consider more similar than those plotted far apart.

## 3 Results

In total, we analysed 147.8 h of sound recordings from 19 nests. Audio files containing each sound type can be found in Appendices S3 and S4, respectively.

Among the six species recorded in the present work, only two of them produced vocalisations: *Podocnemis expansa*, confirming the findings from Ferrara, Vogt, and Sousa‐Lima (2012); and *Batagur baska*. Results from each species are as follows:

### 3.1 *Pseudemydura umbrina* (Siebenrock 1901) (Chelidae)

We analysed 37.5 h of recordings from six nests containing two to four eggs each, being 1 in the wild and 5 in captivity. No sounds were detected over the duration of the recordings, including those in which hatchling were already out of the eggs but still in the nest.

### 3.2 *Podocnemis expansa* (Schweigger 1812) (Podocnemididae)

**FIGURE 1:**
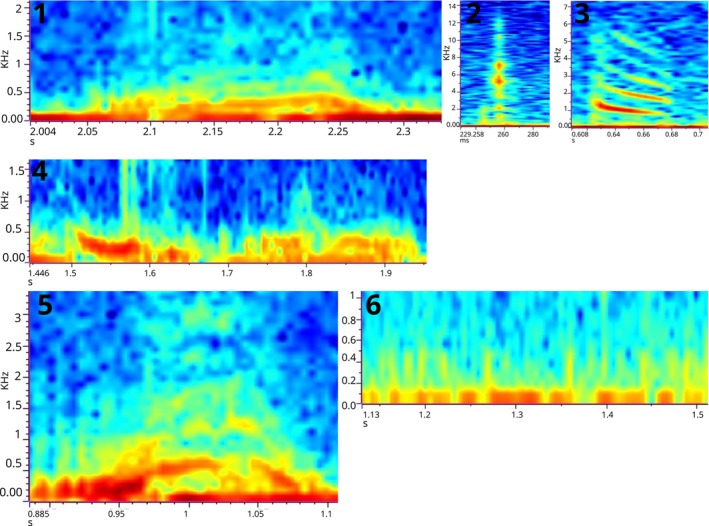
Prehatch acoustic repertoire of *Podocnemis expansa*. Warmer colours represent higher amounts of energy.

In total, from 7 h of recordings of 10 different nests, we were able to identify six different call types (Figure 1). All sounds were produced by both embryos and hatchlings within the nest.

### 3.3 *Chitra indica* (Gray 1831) (Trionychidae)

Over 32 h of recordings were analysed from a subset of the original clutch (42) containing 12 eggs, from which 4 died. We detected cracking sounds, which got more frequent close to hatching date, but no vocalisations were captured. Hatchlings came out of the egg in different dates, with a total difference of 4 days from the first to the last egg. Two of the hatchlings were born alone with over 24 h difference. The other six were born in two groups of three each, also with a day difference.

### 3.4 *Batagur baska* (Gray 1831) (Geoemydidae)

**FIGURE 2:**
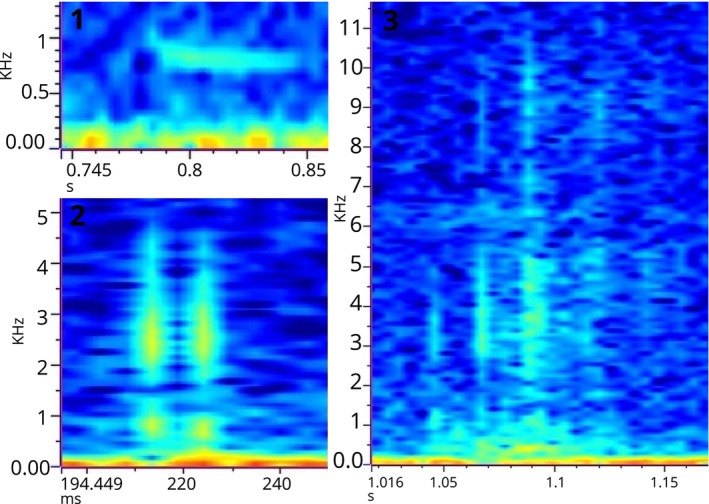
Prehatch acoustic repertoire of *Batagur baska*. Warmer colours represent higher amounts of energy.

In total, we analysed 21.8 h of recordings from one nest originally containing 29 eggs of which 7 hatched. Successful eggs hatched asynchronously, with a total difference of 21 days between the first and the last. We found, in total 22 sounds that were categorised into 3 categories (Figure 2).

### 3.5 *Deirochelys reticularia* (Latreille 1802) (Emydidae)

We analysed 9.5 h of sound recordings from a nest containing 6 eggs from which all survived. Eggs hatched in a relative asynchronous fashion, with a pair of hatchlings popping out every day, with a total difference of 3 days between the first and the last hatched egg. No sounds were found in the recordings except from sparce eggshells cracking.

### 3.6 *Kinosternon subrubrum* (Bonnaterre 1789) (Kinosternidae)

No vocalisations were detected during the 40 h of recordings from one nest containing two eggs. The hatchlings were unable to get out of the egg, so the zoo personnel decided for freeing them out at the same day. Sounds from eggshells cracking got more frequent closer to hatching date.

**FIGURE 3:**
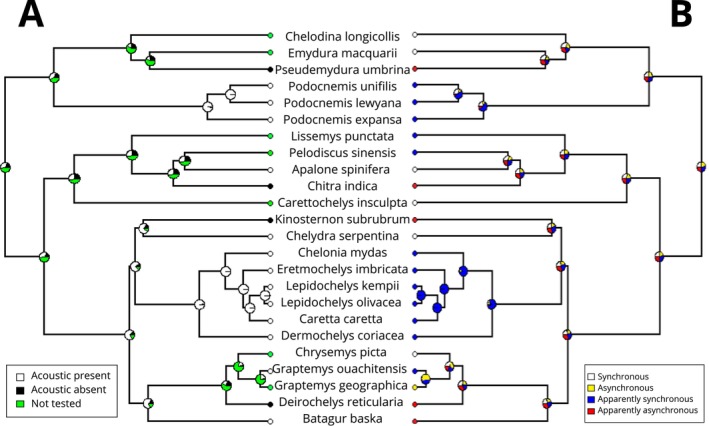
Phylogenetic tree with species of turtles that have been studied regarding within‐nest sound production and/or synchronous behaviour. (A) Information about acoustic behaviour and (B) Information about synchronous behaviour. Both trees include reconstructions of inferred ancestral states (pie charts) in every node.

The character plotting and the ancestral state reconstruction show at least three evolutionary events that culminated in the innovation of within‐egg acoustic behaviour—in podocnemidids, in *Apalone*, and potentially in Durocryptodira (Cryptodira excluding tryonichids). All tree tips reporting presence of vocalisations (13 species representing 7 out 14 turtle families, Figure 3A) match with presence or apparent presence (not formally tested) of synchronous behaviour—except for *Batagur baska*, apparently asynchronous.

**FIGURE 4:**
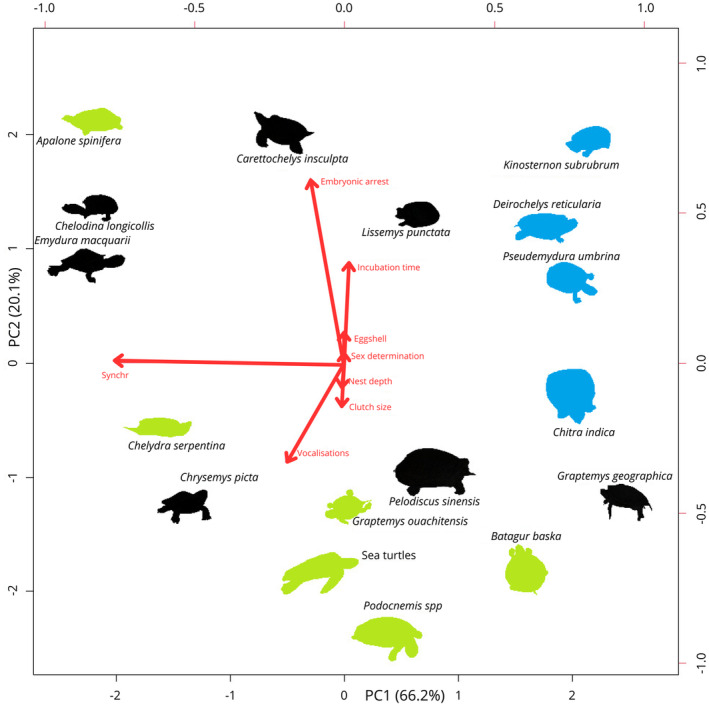
Phylogenetic principal component analysis with traits potentially related to synchronous and vocal behaviour in embryos and hatchlings of a selection of turtle species studied in the present and previous works. Species in green are known to produce sounds form within the egg, in blue are known not to produce any sounds, and in black are not yet studied.

The phylogenetic PCA biplot (Figure 4) indicates that the first PC axis (PC 1) accounts for about 66.2% of the total variance. It is mostly influenced by vectors representing vocalisation and synchronous behaviour. PC2 is mostly influenced by embryonic arrest and incubation time, and accounts for 20.1% of total variance. Detailed results can be seen at Appendix S2.

**FIGURE 5:**
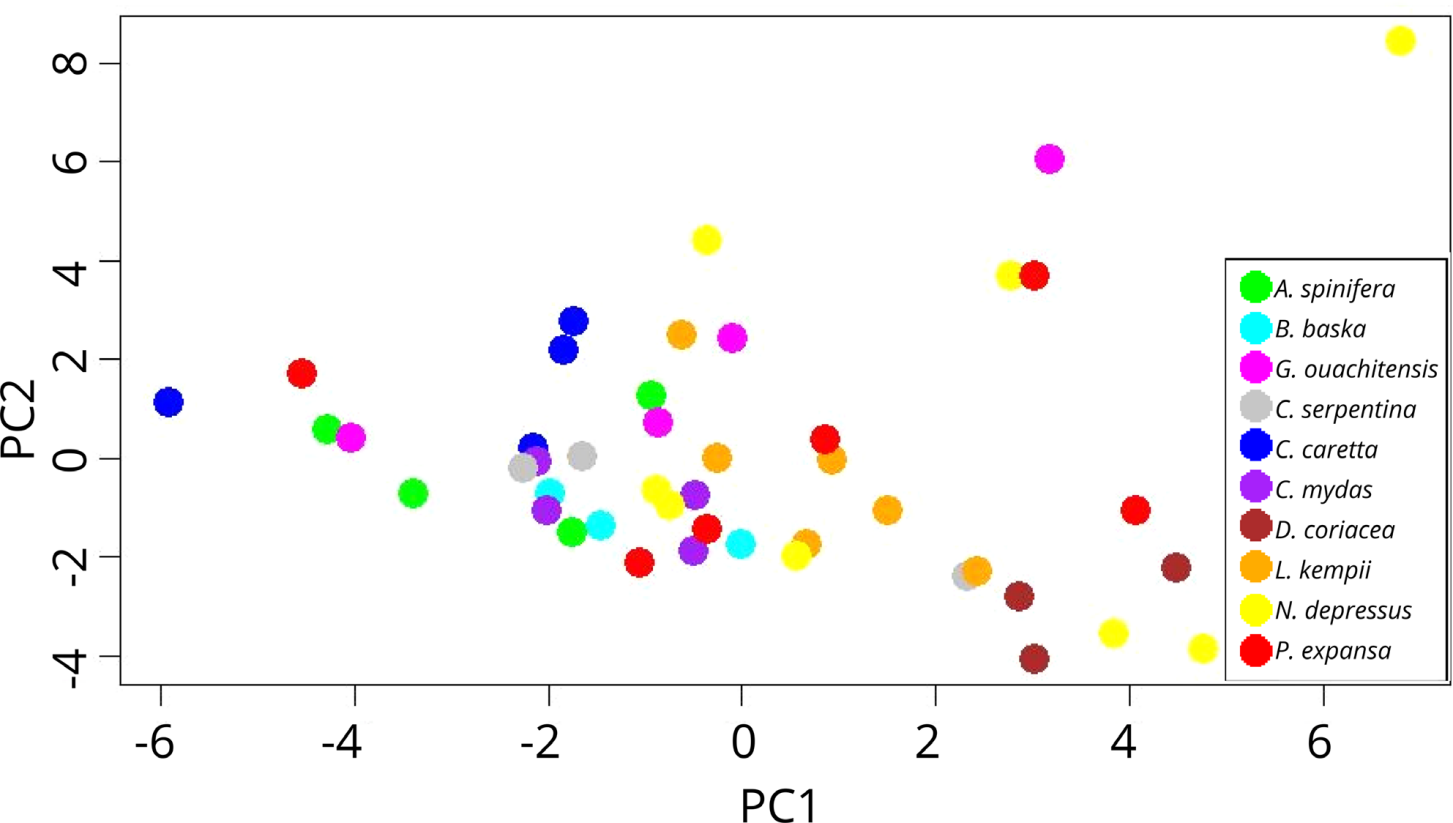
Similarities among within‐egg turtle calls. Dots represent unique call types and colours represent different species.

The PCA based on the spectro‐analysis plotted the points in a seemingly random distribution, indicating lack of phylogenetic signal (Figure 5).

## 4 Discussion

Knowledge about coordinated birth and within‐egg vocalisations is limited to a small number of studied turtle species, and for few of them both variables are reported. Studies that report within‐egg calls focused mostly on sea turtles (Ferrara, Mortimer, et al. 2014; Ferrara, Vogt, et al. 2014; Ferrara et al. 2019; Monteiro et al. 2019; McKenna et al. 2019; Field, McGlashan, and Salmon 2021; Nishizawa et al. 2021; Jorgewich‐Cohen, Townsend, et al. 2022), and species of the Podocnemididae (Ferrara, Vogt, and Sousa‐Lima 2012; Del Río 2022), but also on the Ouachita map turtle (*Graptemys ouachitensis*, Geller and Casper 2019a, 2019b), the common snapping turtle (*Chelydra serpentina*, Lacroix et al. 2022), and the Spiny Softshell Turtle (*Apalone spinifera*, Geller and Casper 2023).

Studies on synchronous hatching behaviour have focused on eight species representing six different families (Spencer, Thompson, and Banks 2001; Colbert, Spencer, and Janzen 2010; Spencer 2012; Doody et al. 2012; McGlashan, Spencer, and Old 2012; McGlashan et al. 2015, 2017; Riley et al. 2020; Field, McGlashan, and Salmon 2021; Bock et al. 2022; Lacroix, Davy, and Rollinson 2022), all of which synchronise birth except for the Northern map turtle (*Graptemys geographica*, Riley et al. 2020). The only species that have been empirically demonstrated to display both behaviours are the loggerhead turtle (*Caretta caretta*, Field, McGlashan, and Salmon 2021) and the common snapping turtle (Lacroix et al. 2021).

The cues used by embryos to alter the incubation time and synchronise hatch are not known at the present moment; various mechanisms may play a role either in isolation or in combination. Since the first cases of within‐egg vocalisations were reported for turtles in the early 2010's, the hypothesis that these sounds are associated to synchronous hatching has been under discussion (Ferrara, Vogt, and Sousa‐Lima 2012; McKenna et al. 2019; Lacroix, Davy, and Rollinson 2022)—especially because embryo vocal communication is widespread among birds and crocodilians (Mariette, Clayton, and Buchanan 2021), and has been shown to mediate synchronous behaviour (Vergne and Mathevon 2008). Nevertheless, the limited number of empirical studies and the lack of data with broad phylogenetic and ecological coverage prevents any interpretations.

In this study, we recorded the clutches of six turtle species that occupy diverse ecological niches and phylogenetic distribution—increasing the knowledge about vocal behaviour to nine families of which six have at least one representative known to vocalise (Podocnemididae, Chelydridae, Cheloniidae, Dermochelyidae, Emydidae and Geoemydidae). Embryos of most species we recorded (4/6), however, did not produce any sounds. The concatenated trees show that the presence of vocal behaviour is associated to the presence (or apparent presence) of synchronous behaviour (Figure 3). This is supported by the phylogenetic PCA biplot, that implies some degree of correlation between the synchronous and vocal behaviour axes (Figure 4).

Our data does not empirically prove if vocalisations mediate social behaviours in embryos and/or hatchlings, but the lack of vocalisations in some species may be insightful. Although it is not possible to prove a negative assumption (i.e., they *do not* vocalise), as it may only reflect the absence of data—Del Río (2022) reported sounds produced by embryos of the Magdalena River turtle (*Podocnemis lewyana*), while Bock et al. (2022) reported not registering any sounds in another study on the same species—our standardised protocol is expected to yield comparable results. If no sounds were produced by most of the recorded species, this indicates, at least, that they are less vocal than the species with positive results.

Excitingly, the existence of seemingly silent embryos challenges the recently proposed idea that within‐nest sounds are no more than accidental subproducts of other behaviours (McKenna et al. 2019; Field, McGlashan, and Salmon 2021). The hypothesis that within‐egg vocalisations mediate social behaviour is supported by the apparent absence of vocalisations in species with small and/or asynchronous clutches, while present in synchronous species with similar ecological niches but different evolutionary histories (i.e., podocnemidids and sea turtles—see more below). Furthermore, the idea that acoustic repertoires composed by several types of sounds play an ecological role is the most parsimonious alternative. Considering that archosaurians are known to mediate synchronous behaviour through within‐egg sounds (Vergne and Mathevon 2008; Mariette, Clayton, and Buchanan 2021), and that vocalisations are generally used in social interactions by adult archelosaurians (turtles + archosaurians; Jorgewich‐Cohen, Henrique, et al. 2022), the role of within‐nest vocalisations should not be dismissed.

Lacroix et al. (2022) got no response in a playback experiment designed to test if sounds induce pipping in eggs of the common snapping turtle. They proposed that either sounds do not mediate synchronous hatching behaviour or that they do so in much more specific and refined manner. In fact, these possibilities are not mutually exclusive if considered in a broad phylogenetic perspective (see below). Whereas focusing on the matter of the potentially higher complexity of sounds mediating within‐nest behaviours, there are several stages from an embryonic stage to the life out of the nest that should be taken into account.

The relevance of acoustic signals potentially starts during the second phase of the embryological development (linked to maturation of the neuromuscular system, whereas the primary is linked to organ/tissue development), a few days before hatching, when neuromuscular activity increases (Spencer, Thompson, and Banks 2001; Colbert, Spencer, and Janzen 2010; McGlashan, Spencer, and Old 2012). At this phase, acoustic signals would possibly play an important role in species that display ‘catch up’ (i.e., *Chelodina longicollis, Emydura mcquarii, Apalone spinifera, Podocnemis lewyana*, and *Caretta caretta*; McGlashan et al. 2015, 2017; Riley et al. 2020; Field, McGlashan, and Salmon 2021; Bock et al. 2022) or ‘delayed’ (not reported in any species so far) synchrony. These types of synchronous behaviours could also be mediated by other channels such as heart rate, vibrations, and chemical cues (Spencer, Thompson, and Banks 2001; Spencer 2012; McGlashan, Spencer, and Old 2012; Mariette, Clayton, and Buchanan 2021), in combination or potentially excluding the use of sounds.

The same or different sounds may stimulate the hitherto mentioned modalities of coordinated synchronous behaviour during pipping and hatching (potentially as two separate events). Additionally, species that present early hatch (i.e., *Chrysemys picta* and *Chelydra serpentina*; Colbert, Spencer, and Janzen 2010; McGlashan, Spencer, and Old 2012) could also benefit from acoustic cues at this stage.

After hatching, sounds could be used to mediate several species‐specific behaviours that can sometimes be classified as apparent or emergence synchrony: both sea turtles and podocnemidids emit sounds while digging (McKenna et al. 2019; Field, McGlashan, and Salmon 2021; present work), a behaviour known to decrease individual energy investment (Rusli, Booth, and Joseph 2016)—especially in species with deep nests (Field, McGlashan, and Salmon 2021). Species that both do and do not necessarily synchronise digging or emergence could be using sounds to mediate waiting periods in the nest (i.e., sea turtles, *Chrysemys*, respectively; Hays, Speakman, and Hayes 1992; McGlashan, Spencer, and Old 2012).

Seemingly, several species that leave the nest *en masse* are known to produce—quite similar—sounds (i.e., sea turtles, podocnemidids, *Dermatemys mawii*; Ferrara, Vogt, and Sousa‐Lima 2012; McKenna et al. 2019; Field, McGlashan, and Salmon 2021; Jorgewich‐Cohen, Townsend, et al. 2022), hypothetically in an ecological strategy that decreases individual risks through predator swamping (Santos et al. 2016), where sounds coming from multiple locations could be helpful to confuse predators, as it is known in other animal groups (Goodale, Ruxton, and Beauchamp 2019). Synchronous nest emergence could also help to avoid exposure in open nests after the exit of clutch mates (Tucker, Paukstis, and Janzen 2008; McGlashan, Spencer, and Old 2012), a behaviour that differs from predator swamping, but can also be sound mediated.

When conducting empirical tests on the role of acoustic cues in embryo and hatchling behaviour, it is crucial that the experimental design takes into account the different phases of development and the different behaviours they may mediate. As much as this approach can lead to clearer correlations between embryos ‘words’ and actions (e.g., Vergne and Mathevon 2008), the outcome can be hard to decipher. McKenna et al. (2019) reported not finding any differences in the sounds produced by embryos and hatchlings of the olive ridley turtles (*Lepidochelys olivacea*) during incubation, hatching, and emerging from the nest. They proposed that these sounds have no biological purpose as they would expect them to differ from each other in each phase—such sounds are, unfortunately, not available.

The lack of more complex vocalisations or a more refined use of specific sounds in association to specific behaviours, emplace of a seemingly random use of an unelaborated repertoire may be a reflex of the developmental stage of hatchling's vocal abilities. Many species are known to babble in the first stages of life, refining their acoustic repertoire later on (i.e., birds, bats, dolphins and humans; Ter Haar et al. 2021; Eggleston et al. 2022). Unfortunately, at present, no studies on the ontogenetic changes of the acoustic repertoire in turtles exist.

Furthermore, comparing putatively sound‐mediated behaviours to (either analogous or homologous) behaviours displayed by potentially mute species can bring several insights on the processes that underlie synchrony. Considering that both synchrony and vocal behaviour have costs (Deecke, Ford, and Slater 2005; Colbert, Spencer, and Janzen 2010), different ecological contexts are expected to yield different combinations of these behaviours.

Some species, in theory, can be synchronous but silent: when behaviours are mediated by other channels of communication, or in cases where synchrony is not embryo coordinated (i.e., environmental and temporal synchrony). Vibro‐acoustic environmental cues such as thunder and rain, and vibrations caused by translocation, can elicit synchronous hatch in the Indian flapshell turtle (*Lissemys punctata*, Vijaya 1983) and the pig‐nosed turtle (*Carettochelys insculpta*, Doody et al. 2012). Experiments at Perth Zoo (unpublished data) have demonstrated that eggs of the Western swamp tortoise (*Pseudemydura umbrina*) have higher chances of hatching when exposed to constant vibrations during incubation—although hatch is asynchronous. In natural context, the pig‐nosed turtle synchronises hatch when the nest gets flooded and embryos experience hypoxia (Doody et al. 2012). The embryos go through a developmental arrest until the rainy season, when conditions are better suitable (Doody et al. 2012). Although embryos of this species have never been sound recorded, our analysis indicates them to be most likely non‐vocal.

We have not found any sounds in the recordings from clutches of the chicken turtle (*Deirochelys reticularia*) and the Eastern mud turtle (*Kinosternon subrubrum*). Both species are asynchronous and also go through diapause (embryological arrest) during incubation (Ewert 1991; Horne 2007)—but without an environmental cue that induces synchronised hatch. Observations from captive breeding suggest that species that go through diapause rarely synchronise hatching (P. Praschag personal observation), which could at least partially explain the lack of vocalisations.

A shorter incubation time (2.5 months or less), with no diapause, is a characteristic in common to all species known to vocalise from within the egg. The Chinese softshell turtle (*Pelodiscus sinensis*), the turtle species with the shortest incubation period (Kuchling 1999), was grouped within the known vocal species in our analysis. In contrast, the common Australian snake‐necked turtle (*Chelodina longicollis*), that can have incubation periods of 2.5 years (Cann 1998), was plotted on the opposite side of the graph. Curiously, studies focused on this species reached opposite conclusions regarding the presence of synchronous hatching (Spencer 2012; McGlashan et al. 2015).

Some species of snake‐necked turtles and mud turtles go through diapause and long incubation periods, although this occurs in the minority of the species in these far‐related genera (Kennett, Georges, and Palmer‐Allen 1993; Booth 2002; Horne 2007). A comparative study on synchronous and acoustic behaviour including species with different ecological traits can help elucidating this matter. Besides turtles, chameleons are the only reptile group in which post‐laying true embryonic diapause is known to exist is some species (Ewert 1991). Like turtles, chameleons display a great diversity of breeding strategies, sometimes performing synchronous birth and/or nest emergence. This fact, together with the recurrent discoveries of ‘mute’ species vocalising, makes chameleon eggs a potential good comparative model to study prehatch calls and synchronous behaviour in reptiles.

Interpreting results from focus species in a phylogenetic perspective can be insightful, but the current widespread absence of data can only lead to preliminary conclusions. The presence of synchronous hatching in two far related species (*Chrysemys picta*, Cryptodira, and *Emydura mcquarii*, Pleurodira) has been used as an argument to propose the plesiomorphy of this trait (Colbert, Spencer, and Janzen 2010; McGlashan, Spencer, and Old 2012). The same authors suggested that the potential ubiquity of synchronous behaviour could explain why the painted turtle (*Chysemys picta*) synchronises hatch although hatchlings overwinter in the nest. The same rationale can be applied to the apparent lack of influence that sounds have over synchronous pipping in the snapping turtle (Lacroix, Davy, and Rollinson 2022). However, our ancestral state reconstruction analysis had no resolution, recovering equal probabilities for all proposed states from both traits in most tree nodes. With the current state of knowledge about synchronous hatch and acoustic behaviour in turtles, it is not possible to infer their ancestral states and, therefore, the homology of these behaviours remains contentious.

Nevertheless, our findings bring new insights about the evolution of synchronous and acoustic behaviours. The production of sounds by embryos of *Batagur baska* can be interpreted as evidence of convergent evolution. Like in the case of sea turtles and podocnemidids, two far‐related groups with similar ecological traits, *B. baska* is a large bodied species that lays soft‐shelled eggs in deep sand nests that incubate during a short period of time (~2 months). Differently from sea turtles and *Podocnemis*, *B. baska* did not synchronise hatch, with some of the eggs from our studied clutch hatching over 20 days apart from each other. We chose to be conservative and treat them as ‘apparently asynchronous’ in our analysis, as there are no published accounts of their behaviour either in the wild or in captivity. Nevertheless, clutches incubated in captivity at the Project Batagur, ran by the Bangladesh Forest Department, hatch within 1 day (P. Praschag personal observation).

Furthermore, egg shell structure may play a role on the efficiency in which sounds travel. All species of which embryos are known to vocalise have soft shelled eggs—we found no sounds in species with hard‐shelled eggs. Among our studied species, *Deirochelys reticularia* was the only species with soft‐shelled eggs that produced no sounds. Soft‐shelled eggs are mostly common in species that lay large clutches in deep nests, a strategy which may help to avoid eggs from breaking during oviposition (Kuchling 1999).

Based on studies that hypothesised that synchrony is as an adaptative behaviour that promotes social facilitation by sharing the costs of digging (Rusli, Booth, and Joseph 2016), Field, McGlashan, and Salmon (2021) proposed that nest depth influences synchrony in nest emergence. Nest depth can potentially induce asynchronous hatchlings in synchronous species (Field, McGlashan, and Salmon 2021) as a consequence of an intense disparity in developmental stages, caused by exposure to different temperatures and the time required to dig out of the nest (e.g., *Chrysemys picta* and *Caretta caretta*; Houghton and Hays 2001; Field, McGlashan, and Salmon 2021). The high degree of nest emergence synchrony observed in some podocnemidids as opposed to *B. baska* and some sea turtles (Houghton and Hays 2001; Rusli and Booth 2016), may be associated with additional environment cues. Rain induces nest emergence in *Podocnemis expansa* (Simoncini et al. 2022)—which could be classified as environmental synchrony (Doody 2011).

Shallow or exposed nests and hard‐shelled eggs of turtles like *Chitra indica, Pseudemydura umbrina* and *Kinosternon subrurbum*, recorded in the present study, may help explain the absence of vocalisations. Temperature gradients among eggs do not change as much in shallow nests and hatching does not seem to be coordinated. Furthermore, these species do not need to invest as much effort in nest emergence as species with deep nests. Many species with small clutches hatch and emerge from nest individually (e.g., *Terrapene ornata* and *Malaclemys terrapin*; Baker et al. 2013), making cooperative digging less important. Additionally, the costs associated with sound production would select for the disappearance of this behaviour in species that do not need to mediate any behaviour—especially in species with single‐egged clutches like the twist‐neck turtle (*Platemys platicephala*) or the pancake tortoise (*Malacochersus tornieri*).

Traits associated to breeding in turtles, such as clutch size, nest depth, eggshell microstructures, egg arrested diapause and synchronous hatching behaviour seem to have evolved convergently and recurrently in the evolutionary history of the group (Ewert 1991; Horne 2007; Jorgewich‐Cohen, Henrique, et al. 2022). Some of these traits seem to be correlated, suggesting convergent evolution selected by similar ecological conditions (Jorgewich‐Cohen, Henrique, et al. 2022). Likewise, synchronous behaviour seemed to have evolved several times in association to species‐specific ecological characteristics. Different types of synchronous behaviours probably have different selective pressures and evolutionary histories, with similar modalities potentially being convergent in different lineages.

Within‐nest vocalisations could have a similar evolutionary pattern to the one observed in synchronous behaviour, potentially having evolved in association. Nevertheless, there is some evidence suggesting embryo calls are most likely a plesiomorphic trait, such as within‐egg vocalisations being widespread in archosaurs and some squamates. Conversely, our phylogenetic distance analysis did not show any patterns based on the phylogenetic distribution of the studied species. This could be an artefact of the limited sample size; or it may indicate that there is no evolutionary pattern associated to such sounds. In the latter case, it can represent both a case of conservative behaviour or a case of strong convergence. Both scenarios rely on the assumption that strong selective pressures (e.g., predation) would maintain or develop similar behaviours in distant lineages. Considering the plethora of findings on the present study, it seems most parsimonious to interpret both within‐egg and synchronous behaviours as traits that converged among lineages with similar ecologies. Understanding the mechanisms that mediate synchronous behaviours may help elucidating this mystery.

## 5 Conclusions

Communication is central to group mediation and sociality. There are many social behaviours expressed by turtles during development, from embryo to nest emergence, that could be mediated by acoustic signals. Synchronous birth might not necessarily be coordinated by sounds in every species—as it seems to be the case in *Chelydra serpentina* (Lacroix, Davy, and Rollinson 2022)—but may be important for others. It is crucial that more experiments are conducted combining synchrony and acoustic tests, so we can have a clearer understanding of the patterns in which these behaviours are associated. Moreover, future work should aim to understand the behavioural patterns of synchronous embryonic development, hatch, dig, nest emergence, and dispersal as separate ecological events, as sounds might be used to mediate one of these behaviours but not the other.

## Author Contributions

**Gabriel Jorgewich‐Cohen:** conceptualization (equal), data curation (equal), formal analysis (equal), funding acquisition (equal), investigation (equal), methodology (equal), project administration (equal), resources (equal), software (equal), validation (equal), visualization (equal), writing – original draft (equal), writing – review and editing (equal). **Madeleine Wheatley:** data curation (equal), methodology (equal), writing – original draft (equal). **Lucas Pacciullio Gaspar:** data curation (equal), formal analysis (equal), investigation (equal), software (equal). **Peter Praschag:** data curation (equal), investigation (equal), project administration (equal), supervision (equal). **Nicole Scholte Lubberink:** data curation (equal), formal analysis (equal), investigation (equal), software (equal), visualization (equal). **Keesha Ming:** data curation (equal), software (equal). **Nicholas A. Rodriguez:** data curation (equal), investigation (equal), resources (equal). **Camila R. Ferrara:** data curation (equal), investigation (equal), supervision (equal), writing – original draft (equal).

## Conflicts of Interest

The authors declare no conflicts of interest.

## Supporting information


Appendix S1

Appendix S2

Appendix S3

Appendix S4

Appendix S5

## Data Availability

The data that support the findings of this study are available on [Link], [Link], [Link], [Link], [Link] and on Dryad. Any further detail that might be requested will be fully supplied by the corresponding author.
